# Interhemispheric Functional Connectivity and Its Relationships with Clinical Characteristics in Major Depressive Disorder: A Resting State fMRI Study

**DOI:** 10.1371/journal.pone.0060191

**Published:** 2013-03-29

**Authors:** Li Wang, Ke Li, Qing-E Zhang, Ya-Wei Zeng, Zhen Jin, Wen-Ji Dai, Yun-Ai Su, Gang Wang, Yun-Long Tan, Xin Yu, Tian-Mei Si

**Affiliations:** 1 Institute of Mental Health, Peking University, Beijing, China; 2 The Key Laboratory for Mental Health, Ministry of Health, Beijing, China; 3 Department of Radiology, 306 Hospital of People’s Liberation Army, Beijing, China; 4 Mood Disorders Center, Beijing Anding Hospital, Capital Medical University, Beijing, China; 5 Center for Psychiatric Research, Beijing Huilongguan Hospital, Beijing, China; Hangzhou Normal University, China

## Abstract

**Background:**

Abnormalities in large-scale, structural and functional brain connectivity have been increasingly reported in patients with major depressive disorder (MDD). However, MDD-related alterations in functional interaction between the cerebral hemispheres are still not well understood. Resting state fMRI, which reveals spontaneous neural fluctuations in blood oxygen level dependent signals, provides a means to detect interhemispheric functional coherence. We examined the resting state functional connectivity (RSFC) between the two hemispheres and its relationships with clinical characteristics in MDD patients using a recently proposed measurement named “voxel-mirrored homotopic connectivity (VMHC)”.

**Methodology/Principal Findings:**

We compared the interhemispheric RSFC, computed using the VMHC approach, of seventeen first-episode drug-naive patients with MDD and seventeen healthy controls. Compared to the controls, MDD patients showed significant VMHC decreases in the medial orbitofrontal gyrus, parahippocampal gyrus, fusiform gyrus, and occipital regions including the middle occipital gyrus and cuneus. In MDD patients, a negative correlation was found between VMHC of the fusiform gyrus and illness duration. Moreover, there were several regions whose VMHC showed significant negative correlations with the severity of cognitive disturbance, including the prefrontal regions, such as middle and inferior frontal gyri, and two regions in the cereballar crus.

**Conclusions/Significance:**

These findings suggest that the functional coordination between homotopic brain regions is impaired in MDD patients, thereby providing new evidence supporting the interhemispheric connectivity deficits of MDD. The significant correlations between the VMHC and clinical characteristics in MDD patients suggest potential clinical implication of VMHC measures for MDD. Interhemispheric RSFC may serve as a useful screening method for evaluating MDD where neural connectivity is implicated in the pathophysiology.

## Introduction

Abnormalities in large-scale, brain connectivity have been increasingly reported in patients with major depressive disorder (MDD). During various task-directed processes, such as working memory [1], executive control [2], facial emotion recognition [3], and reward processing [4], depressed individuals exhibited significantly disturbed connectivity between the task-related regions. Further, resting state studies have shown specific network alterations in MDD patients, typically involving the default mode network [5] and the cognitive control network [6]. However, MDD-related alterations in the functional interaction between the cerebral hemispheres are still pooly understood.

Convergent evidence from normal and split-brain studies has highlighted the importance of interhemispheric coordination to human behaviors. Many earlier studies suggest that communication between the left and right cerebral hemispheres is a crucial component of cognitive and emotional processing [7]–[9]. Furthermore, interhemispheric coordination is especially needed for the execution of complex tasks [10]–[11]. When work load or task complexity increases, more interhemispheric cooperation is required [10]–[11]. Additionally, split-brain studies has demonstrated that humans with sectioned corpus callosum (CC, the largest white-matter connection between the hemispheres) had deficits in the sensory, motor, and language processing [12]–[14] as well as cognitive function, such as impairment in attentional maintenance [15]–[16]. Based on the importance of bi-hemispheric coordination for the emotions and higher-order cognitive functions, and the core emotional symptoms that are often accompanied by impairment in a series of cognitive functions in MDD [17], it is reasonable to expect some deficits in interhemispheric interaction in MDD patients.

Indeed, evidence from both structural and microstructural studies has strongly implicated the presence of interhemispheric interaction deficits in MDD. The corpus callosum, the major white-matter tract connecting the two hemispheres, plays a critical role in the interhemispheric communication, particularly in the integration of emotional, higher-order cognitive, and perceptual processing [18]–[19]. Structural magnetic resonance imaging (MRI) studies have demonstrated area and shape changes in the CC of patients with MDD [20]–[22]. Furthermore, diffusion tensor imaging (DTI) studies reported lower fractional anisotropy (FA) in the genu [23]–[24] and posterior body [25] of the CC in MDD patients. Abnormalities in the CC, together with widely impaired white-matter integrity in the frontal, temporal, and parietal lobes of MDD patients [26], may affect interhemispheric functional coordination, thereby contributing to the emotional and cognitive symptoms of MDD.

Preliminary evidence from functional imaging studies has suggested impaired functional coordination between two hemispheres in MDD. Earlier studies using sleep electroencephalography (EEG) have implicated reduced interhemispheric coordination in MDD, from evidence of a reduced coherence of the beta and theta frequency bands between two hemispheres reported in patients with acute [27] and remitted [28] depression and individuals at genetic risk for depression [29]. Moreover, studies using positron-emission tomography (PET) and functional MRI (fMRI) have demonstrated imbalanced activity in homologous regions between hemispheres in MDD, particularly in the prefrontal cortex. Left-hemisphere hypoactivity [30] and right-hemisphere hyperactivity [31] have been reported in the dorsal lateral prefrontal cortex (DLPFC) of MDD patients and, an imbalance between activity of the left and right DLPFC was found to be linked to a negative emotional bias seen in MDD [32].

Resting state fMRI (R-fMRI), which reveals the patterns of coherent spontaneous fluctuations of blood oxygen level dependent (BOLD) signals [33], provides a novel approach for examining the interhemispheric interaction. Functional homotopy, defined as the high degree of synchrony in spontaneous activity between geometrically corresponding interhemispheric regions, is a basic principle of the brain's intrinsic functional architecture [34]. Typically, most of the functional networks identified using task-based fMRI are bilaterally distributed [9], [35]. Studies [36]–[37] using R-fMRI have revealed robust patterns of correlated spontaneous activity between homologous regions in opposite hemispheres, along with systematic regional variations, consistent with models of brain's functional hierarchy [37]. As a salient feature of brain's functional architecture, homotopic RSFC may be a sensitive index for detecting alterations of interhemispheric interaction in MDD.

Here, we directly evaluated homotopic RSFC in patients with first-episode drug-naive MDD using a recently validated approach named “voxel-mirrored homotopic connectivity (VMHC; [38])”. VMHC measures the RSFC between each voxel in one hemisphere and its mirrored counterpart in the opposite hemisphere. Using the VMHC method, abnormal homotopic RSFC has been demonstrated in schizophrenia [39], autism [40], and cocaine addiction [41]. Based on the findings of prior structural, microstructural, and functional imaging studies of MDD, we hypothesized that an impairment of interhemispheric functional coordination may be involved in the pathogenesis of MDD, which would be reflected as reduced homotopic RSFC in MDD patients. Moreover, given the importance of bilateral hemispheric coordination for the emotional and cognitive functions, some clinical relevancies of VMHC measure were also expected.

## Materials and Methods

### Ethics Statement

This research protocol was approved by the Ethics Committee in the Peking University Institute of Mental Health and the Ethics Committee of the Beijing Anding Hospital of Capital Medical University.

### Subjects

18 patients with first-episode MDD were recruited from the Peking University Institute of Mental Health and Beijing Anding Hospital of Capital Medical University between May 2010 and July 2011. A diagnosis of first-episode MDD was made by two psychiatrists using the Diagnostic and Statistical Manual fourth edition (DSM-IV) criteria for MDD [17]. Inclusion criteria included a current acute episode of depression; drug naive, unipolar subtype; a total score on the 17-item Hamilton Depression Rating Scale (HDRS) [42] of no less than 24; and a duration of depression ≥1 months but ≤24 months. The specific exclusion criteria was other diagnoses within the past year, including organic mental disorders, schizophrenia, schizoaffective disorder, psychotic features either congruent or incongruent with mood, bipolar disorder, and substance dependence and abuse. On the day of scanning, the symptoms of patients were assessed using the 17-item HDRS and Hamilton Anxiety Rating Scale (HAMA) [43].

18 age-, gender-, and education-matched control subjects were recruited from the local community. The controls with a HDRS score more than 7, any current or lifetime psychiatric or neurological disorders, and a family history of major psychiatric or neurological illness in their first-degree relatives were excluded.

Exclusion criteria for all the subjects included the following: age under 18 or above 60; any unstable medical condition, neurological disorders, and history of significant head trauma; current or previous use of antidepressant or antipsychotic drugs; a history of electroconvulsive therapy; acutely suicidal or homicidal behaviors; current pregnancy or breastfeeding; and contraindications to a MRI scan.

All of the subjects were right-handed as determined by the Edinburgh Handedness Scale [44]. Before entering the study, all participants signed written informed consent as approved by the local Institutional Review Boards. Each individual was compensated for their participation in our study. One of the patients converted to a manic episode in one and a half year after the fMRI scan. So this patient was excluded and the data of 17 ones left were used in this study.

### MRI Data Acquisitions

The images were acquired with a 3.0 T Siemens Tim Trio whole-body MRI system (Siemens Medical Solutions, Erlangen, Germany) at the Department of Radiology of the 306th Hospital of the People’s Liberation Army. First, a structural scan was acquired for the localization of functional scans. Then, the functional images were recorded axially over 7 min and 6 s using an echo-planar imaging (EPI) sequence with the following parameters: repetition time (TR)/echo time (TE) = 2000/30 ms, 90° flip angle, 30 slices, slice thickness/gap = 4.0/0.8 mm, voxel size = 3.3×3.3×4.0 mm^3^, resolution = 64×64 matrix, field of view (FOV) = 210 mm×210 mm, and band-width = 2232 Hz/pixel. After that, the three-dimensional T1-weighted magnetization prepared rapidly acquired gradient echo (MPRAGE) images were acquired sagittally using the following parameters: TR/TE = 2300/3.01 ms, resolution = 256×256 matrix, slice thickness = 1 mm, 176 slices, FOV = 256 mm×240 mm, voxel size = 1×1×1 mm^3^, 9° flip angle. During the resting state scans, the subjects were instructed to keep their eyes closed, remain still, and not think of anything in particular.

### Functional Image Preprocessing

The preprocessing was performed using the Data Processing Assistant for Resting-State fMRI (DPARSF) [45] with statistical parametric mapping (SPM8, http://wwwfil.ion.ucl.ac.uk/spm). The first ten volumes were discarded to allow for scanner calibration and adaptation of the participants to the scanning environment. The remaining 200 volumes were analyzed. The steps included slice timing, head-motion correction, spatial normalization in the Montreal Neurological Institute (MNI) space using the transformation parameters estimated using an unified segmentation algorithm [46] (see the following “Structural Image Analysis” section for details) and re-sampling with a 3×3×3 mm^3^ resolution. The generated images were smoothed with a Gaussian kernel of 6 mm at full-width at half-maximum (FWHM). Subsequently, to reduce the spurious BOLD variances unlikely to reflect neuronal activity [33], [47], several sources of spurious variance were removed, including the six motion parameters obtained by head-motion correction, linear drift, signals from the ventricular system, white matter, and whole brain. These masks were acquired using a priori probability template provided by SPM8, and thresholded at 0.7 (ventricular mask), 0.9 (white matter mask), and 0.5 (whole brain mask). The residuals of these regressions were band-pass filtered (0.01–0.08 Hz) to reduce low-frequency drift and high-frequency noise. After filtering, the images of each subject were registered to a study-specific symmetric MNI template (see the “Structural Image Analysis” section for details) and were then used to compute the homotopic RSFC.

### Voxel-Mirrored Homotopic Connectivity

The VMHC computation was performed using DPARSF software. For each subject, the homotopic RSFC was computed as the Pearson correlation coefficient between each voxel’s residual time series and that of its symmetrical interhemispheric counterpart. Correlation values were then Fisher z-transformed to improve normality. The resultant values constitute the VMHC and were used for the group analyses.

### Structural Image Analysis

To exclude the influence of structural damage to VMHC measures, we examined whether there were abnormalities in the gray-matter volumes of the regions showing altered VMHC. A voxel-based morphometry (VBM) analysis was performed to compute the gray-matter volume of each subject using DPARSF software. Briefly, individual T1 images were co-registered to the mean functional images after head-motion correction. The transformed images were segmented into gray matter, white matter, and cerebrospinal fluid and were then normalized to the MNI space using an unified segmentation algorithm [46]. After that, the registered gray-matter images were modulated and smoothed with a 6-mm Gaussian kernel. Additionally, the normalized gray-matter images of all subjects were averaged, and the generated mean image was averaged with its left-right mirror to get the symmetrical template and mask for VMHC and statistical analyses.

### Statistical Analysis

First, to examine the differences in VMHC between patients and controls, the individual VMHC maps were entered into a voxel-wise two-tailed t-test. The result was thresholded using *p*<0.01 (|*Z*|>2.58) for each voxel and minimum cluster size of 810 mm^3^, resulting in a corrected *p*<0.05, as determined by a Monte Carlo simulation (see AlphaSim in AFNI http://afni.nimh.nih.gov/pub/dist/doc/manual/AlphaSim.pdf; parameters were: single voxel *p* = 0.01, 6-mm FWHM, with the unilateral hemispheric gray-matter mask (there is only one correlation for each pair of homotopic voxels)).

Then, to observe the clinical relevancies of VMHC, we computed Pearson's correlation coefficients between the VMHC and each clinical characteristic of the MDD patients in a voxel-wise manner. These analyzed clinical variables included illness duration, total HDRS score and symptom factors of HDRS (including anxiety, weight loss, cognitive disturbance, retardation, and sleep disturbance). The threshold was set at a corrected *p*<0.05, estimated using the same parameters as the group comparison analysis of VMHC.

Given that a prior study has suggested that functional connectivity at rest could be affected by micromovements from volume to volume [48], we calculated the framewise displacement (FD) values for each subject, which can reflect the temporal derivative of the movement parameters. One control subject who had FD>0.5 mm on more than 35 volumes was excluded from the group-level analyses. The mean FD was added as covariates in the group statistical analyses of VMHC.

Complementarily, we extracted the mean values of gray-matter volume in regions showing group differences in VMHC, and then these values for each region were compared between patients and controls using two sample t-tests both with and without Bonferroni correction. For all of the above analyses, age and gender were added as covariates.

## Results

### Sample Characteristics

As shown in Table 1, there were no significant differences between MDD patients and healthy controls in gender, age, or years of education. The maximum of head movements of all subjects were less than 1 mm in any translation direction and 1° in any angular dimension. No significant differences were found in maximum movement values in each plane of translation (x: *t* = -.25, *p* = .81; y: *t* = 1.03, *p* = .31; or z: *t* = .25, *p* = .80) or each plane of rotation (pitch: *t* = .33, *p* = .72; roll: *t* = .36, *p* = .72; or yaw: *t* = 0.55, *p* = 0.59) between the two groups. No significant difference (*t* = .29, *p* = .78) was found in FD values between patients (Mean±standard deviation [SD]: 0.23±0.15 mm) and controls (0.22±0.09 mm).

**Table 1:** Demographics and clinical characteristics of the subjects.

Variable (Mean±SD)	MDD (*n = *17)	HC (*n = *17)	*p* value
Gender (male/female)	8/9	9/8	0.73^a^
Age (year)	34.00±13.29	34.82±12.22	0.85^b^
Education (year)	13.00±2.35	13.35±2.37	0.66^b^
Illness duration (month)	6.75±7.26	–	
Total HDRS score	26.58±3.43	3.30±2.65	<0.01^b^
Anxiety	7.94±2.07	0.65±0.59	<0.01^b^
Weight loss	1.24±0.75	0.00±0.00	<0.01^b^
Cognitive disturbance	5.65±1.50	0.60±0.65	<0.01^b^
Retardation	7.59±1.70	1.02±0.90	<0.01^b^
Sleep disturbance	4.12±1.65	1.03±0.93	<0.01^b^
Total HAMA score	18.18±6.59	–	

MDD, Major Depressive Disorder; HC, Healthy Control.

a and ^b^ indicate the *p* values for the chi-squared test and two-sample t-test, respectively.

### VMHC Differences between Groups

**Figure 1:**
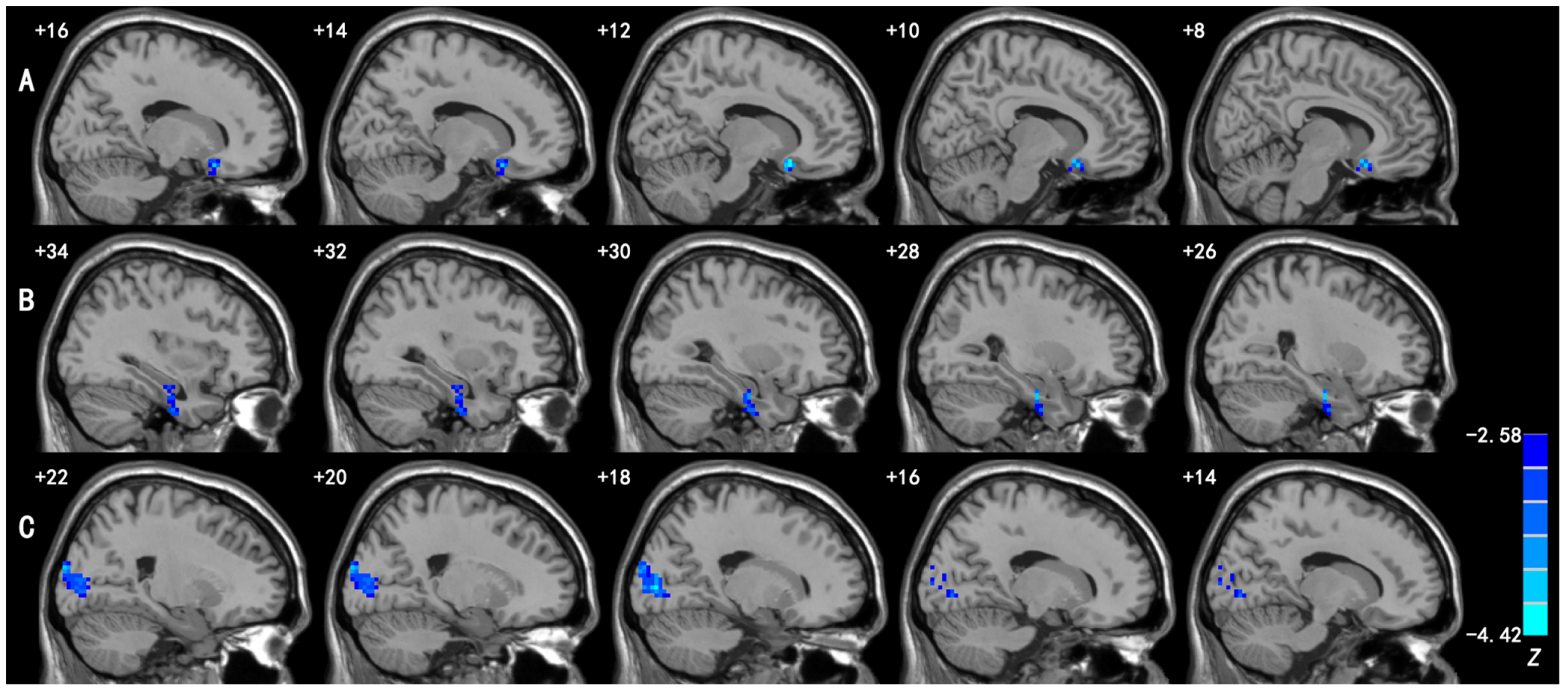
Regions showing significant differences in VMHC between MDD patients and healthy controls. Blue colors indicate reduced VMHC in patients compared to the controls. A: medial orbitofrontal gyrus; B: parahippocampal gyrus/fusiform gyrus; and C: middle occipital gyrus/cuneus. The threshold was set at a corrected *p*<0.05. Slices are 2 mm apart. The numbers at the top left of the map refer to the x-coordinates in the MNI space. The *Z*-score bar is shown at the lower right of the figure.

Compared to the controls, the MDD patients showed significant decreases in VMHC, but no increase, in the medial orbitofrontal gyrus, parahippocampal gyrus/fusiform gyrus, and occipital regions including the middle occipital gyrus and cuneus. See Figure 1 and Table 2 for details. Before and after Bonferroni correction, no significant changes were found in the gray-matter volumes of these regions showing abnormal VMHC in MDD patients.

**Table 2:** Regions showing significant differences in VMHC between MDD patients and healthy controls.

Region	BA	Cluster size (mm^3^)	*Z* score	MNI coordinates(x, y, z)
Medial orbitofrontal gyrus	11	1053	−3.647	±12, 18, −18
Parahippocampal gyrus/fusiform gyrus	35/37	1512	−4.148	±24, −12, −27
Middle occipital gyrus/cuneus	18	3348	−4.158	±18, −87, 3

BA: Brodmann's area; *Z*: statistical value of the peak voxel; x, y, z: coordinate of primary peak locations in the MNI space.

### Relationships between VMHC and Clinical Variables

**Figure 2:**
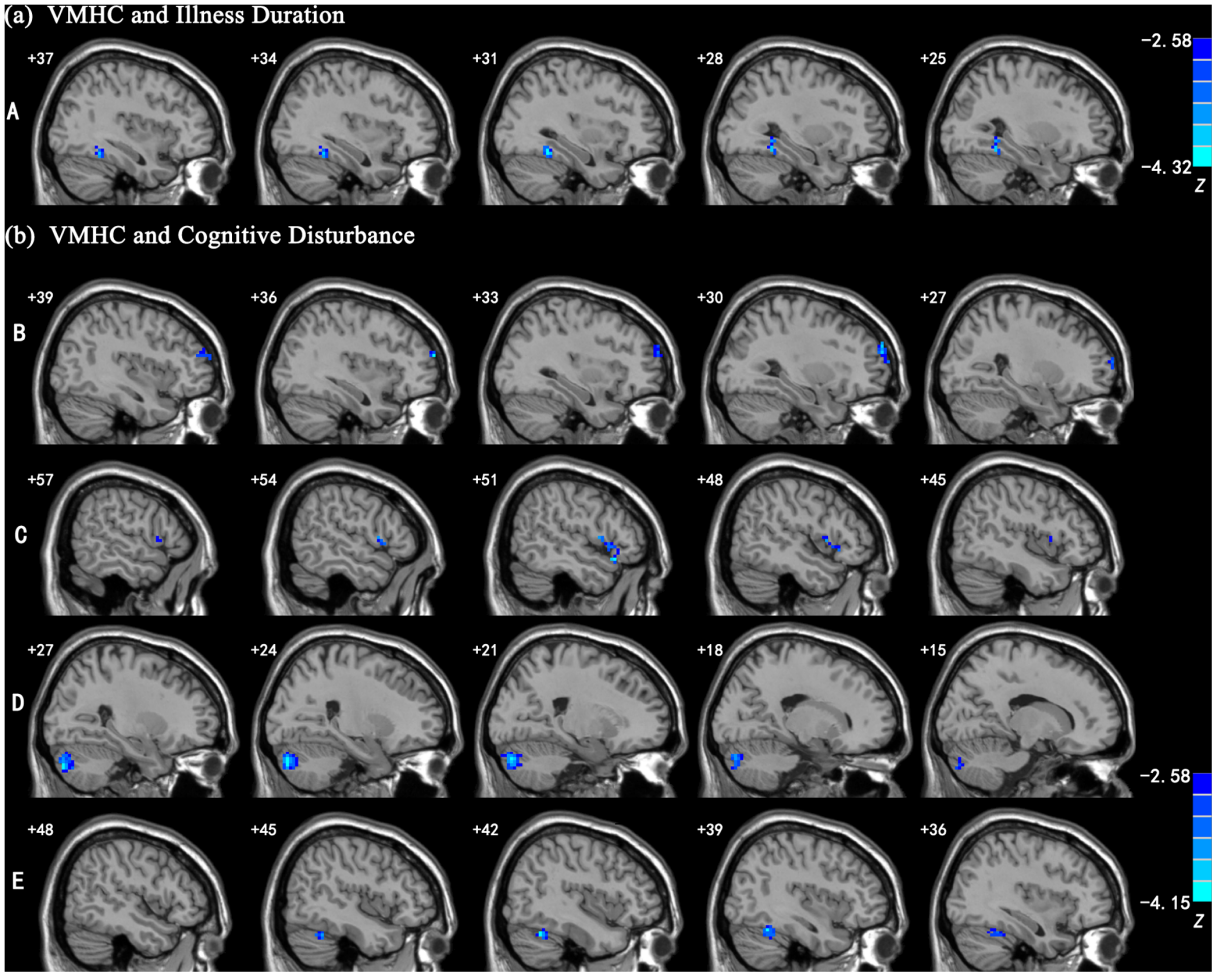
Regions showing significant correlations between VMHC and clinical variables in MDD patients. (1): the region showing significant correlation between the VMHC and illness duration; (2): the regions showing significant correlations between the VMHC and severity of cognitive disturbance. Blue colors indicate negative correlations. A: fusiform gyrus; B: middle frontal gyrus; C: inferior frontal gyrus; D: cerebellum crus_2; and E: cerebellum_6. The threshold was set at a corrected *p*<0.05. Slices are 3 mm apart. The numbers at the top left of the map refer to the x-coordinates in the MNI space. The Z-score bars are shown at right of the figure.

There was a significant negative correlation between the illness duration and VMHC of the fusiform gyrus. Significant negative correlations were also found between the severity of cognitive disturbance and VMHC in the prefrontal regions, including the middle and inferior frontal gyri, and cerebellar regions, such as cerebellum crus_2 and cerebellum_6. See Figure 2 and Table 3 for details.

**Table 3:** Regions showing significant correlations between VMHC and clinical variables in MDD patients.

Clinical variable	Region	BA	Cluster size (mm^3^)	*Z* score	MNI coordinates(x, y, z)
Illness duration	Fusiform gyrus	37	918	−3.825	±27, −48, −12
HDRS-Cognitive disturbance	Middle frontal gyrus	10	1404	−3.830	±36, 60, 18
	Inferior frontal gyrus	47	918	−3.702	±51, 21, −15
	Cerebellum crus_2	–	4212	−4.011	±21, −84, −33
	Cerebellum_6	–	1215	−4.112	±39, −54, −24

BA: Brodmann’s area; *Z*: statistical value of the peak voxel; x, y, z: coordinates of primary peak locations in the MNI space.

## Discussion

The primary findings of this study are: (1) MDD patients showed significant decreases in the homologous RSFC in regions including the medial orbitofrontal gyrus, parahippocampal gyrus, fusiform gyrus, and the occipital regions. These results are in agreement with the above-reviewed studies showing alterations of interhemispheric interaction in MDD; (2) there was also some evidence to suggest inverse relationships between the degree of cognitive disturbance and VMHC in the prefrontal and cerebellar regions, and between the illness duration and VMHC of the fusiform gyrus in MDD patients. These observed group differences and clinical relevancies of VMHC may have important functional implications for MDD.

We found a reduced interhemispheric RSFC in the medial orbitofrontal gyrus (mOFG) of MDD patients. The mOFG exerts top-down inhibition on subcortical regions, such as the amygdala, thereby participating in the regulation of emotion [49]. Earlier studies found that interhemispheric communication is facilitated for emotionally significant compared to neutral stimuli in healthy individuals [8], [50]. In MDD patients, disturbed activity and connectivity in the mOFG have frequently been reported during rest [51]–[52] and task-related processes [53]. Whereas prior studies have demonstrated intrahemispheric functional abnormality of the mOFG, indirect evidence from structural and microstructural investigations of the corpus callosum suggests that interhemispheric functional interaction in this region may also be altered in MDD. Impairment in the genu of CC, the major white-matter tract connecting homologous regions of the left and right prefrontal cortices including the mOFC, has commonly been reported in MDD [23]–[24]. Moreover, a DTI study has revealed elevated left and reduced right medial orbitofrontal FA in patients with bipolar depression [54]. Based on the above-mentioned and our current findings, it is proposed that interhemispheric functional coordination in the mOFG may be disturbed in MDD patients and, may lead to a weakening in the bi-hemispheric processing advantage for the emotional information, thus mediating the emotional symptoms of MDD.

We also observed a decrease in interhemisphere RSFC of the parahippocampal gyrus (PHG) in MDD patients. Located around the hippocampus, the PHG plays an important role in memory and emotional regulation [55]–[56]. Abnormal activity in the PHG has been observed in MDD patients during resting state [57]–[58] and task-related processes such as memory [59] and negative emotion processing [53]. Moreover, reduced white-mater integrity in the right PHG has been reported in adults with first-episode drug-naive MDD [60]. Whereas the findings of previous studies have implicated the involvement of the PHG in the neural mechanism of MDD, our study provides new evidence for alterations in the interhemispheric connectivity of the PHG in MDD patients. Given the roles of the PHG in memory and emotional processing, we speculate that reduced RSFC between the bilateral PHG of MDD patients may affect the functional coordination of this region during related task processes, thereby contributing to the emotional symptoms and memory deficits observed in MDD [61].

Other areas that showed reduced interhemispheric RSFC in MDD patients included the fusiform gyrus and the occipital regions such as middle occipital gyrus and cuneus. These regions are within the visual recognition network [51], and are involved in the perception of facial emotion [62], which is crucial for social functioning. Depression has been associated with both structural and functional alterations in these regions, such as decreased gray-matter volume in the cuneus [63] and decreased activity in the left middle occipital and fusiform gyri [64]. Evidence from first-episode drug-naive MDD patients has demonstrated decreased nodal centralities [58] and white-matter integrity [26] related to occipital regions. Our results suggest that MDD patients have impaired interhemispheric coordination in the visual recognition regions, which we speculate to be a potential neural mechanism underlying the deficits of facial emotion processing observed in MDD [65]. Indeed, altered responsiveness to facial emotional stimuli has been used as a biomarker for the early diagnosis of MDD [66]. Thus, our results, together with the previous findings showing intrahemispheric alterations, strongly suggest the involvement of the visual perception regions in the pathogenesis of MDD.

Further analyses in the MDD patients demonstrated some significant correlations between the VMHC and clinical measures, although these clusters did not overlap the regions showing altered VMHC. A negative correlation was found between the duration of depression and VMHC in the fusiform gyrus, suggesting that this region may have a role in the chronicity of MDD. Supporting this, a newly published study found an inverse correlation between the regional homogeneity of left fusiform gyrus and illness duration in MDD patients [67]. Given that all patients in this study were in their first depressive episode with relatively short disease durations, this hypothesis needs to be confirmed in patients with longer illness duration. Moreover, we observed significant inverse correlations in the degree of cognitive disturbance assessed using HDRS and VMHC of the prefrontal regions, including the middle and inferior frontal gyri, and the cerebellum. Both the prefrontal and cerebellar regions are devoted to a variety of cognitive functions, such as planning and executive control [68]–[70]. These types of higher-order cognitive functions may have a bi-hemispheric processing advantage based on supporting evidence that interhemispheric coordination is particularly important for the performance of complex tasks [10]. Therefore, it is easy to understand the inverse relations between VMHC of the prefrontal and cerebellar regions and the cognitive disturbances observed in our patients, suggesting that VMHC measurements in these regions may be used as indicators for the cognitive impairment of MDD.

It is of interest to speculate on the potential underlying mechanisms of the VMHC deficits which we have demonstrated. Our VBM analysis revealed no significant gray-matter changes in any of the regions showing altered VMHC in MDD patients. It therefore seems unlikely that the observed changes in the VMHC were caused by a reduction in tissue volume. We speculated that widespread impairment in the white-matter integrity of MDD patients, particularly in the corpus callosum, may be a likely mechanism; however, it is important to note that, studies on split-brain patients have found that a normal complement of resting state networks and intact functional coupling between hemispheres can emerge in the absence of the corpus callosum [71]–[72]. These findings suggest that normal interhemispheric functional connectivity may arise from more complex pathways, such as common subcortical drivers or complex network-level synchronization, neither of which require direct structural connectivity between cortical components. Therefore, elucidating the mechanism underlying the VMHC alterations in MDD will require combined functional and structural studies. More crucially, a clearer understanding of the large-scale connectivity mechanisms between the two hemispheres is needed.

There are several limitations in the present study. First, the sample size was relatively small; thus, the findings should be considered preliminary and need to be replicated in a larger sample. Second, the brain is not symmetric. However, we tried to resolve this issue by using a symmetric template and by smoothing the functional data. Third, as discussed above, the structural basis underlying the VMHC deficits is unknown. Future studies using a combined analysis of multimodal imaging data would provide direct information on the structural and temporal aspects of interhemispheric interaction in the same subjects. Fourth, these current results were obtained under resting condition. Future studies should combine both resting and task-based fMRI to observe whether the interhemispheric connectivity in currently reported regions is also disturbed under task conditions. Additional neuropsychological tests are also needed to verify our speculations about specific behavioral associations of VMHC deficits. Finally, due to the cross-sectional design, whether these regions with abnormal VMHC change dynamically after therapy needs to be explored in future longitudinal studies.

In summary, we found reduced interhemispheric functional connectivity in patients with MDD during resting state. The findings suggest that functional coordination between the two hemispheres is impaired in MDD patients, thereby providing new evidence supporting a disturbance in long-range, interhemispheric connections in MDD. Furthermore, the inverse relations between the cognitive disturbance and VMHC of the prefrontal and cerebellar regions and between the illness duration and VMHC of the fusiform gyrus observed in our patients suggest potential clinical implication of VMHC measure for MDD. Finally, interhemispheric RSFC may serve as a useful screening method for evaluating MDD where neural connectivity is implicated in the pathophysiology.
